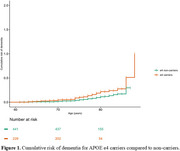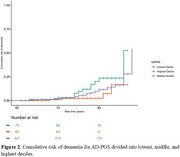# Genetic risk for Alzheimer's disease is associated with dementia in patients with bipolar disorder

**DOI:** 10.1002/alz70860_105440

**Published:** 2025-12-23

**Authors:** Jenna Najar, Robert Sigström, Lina Jonsson, Mikael Landén

**Affiliations:** ^1^ Alzheimer Center Amsterdam, Neurology, Vrije Universiteit Amsterdam, Amsterdam UMC location VUmc, Amsterdam, Netherlands; ^2^ Psychiatry and Neurochemistry, Sahlgrenska Academy, University of Gothenburg, Gothenburg, Sweden; ^3^ Genomics of Neurodegenerative Diseases and Aging, Human Genetics, Amsterdam UMC, Amsterdam, Netherlands

## Abstract

**Background:**

Individuals with bipolar disorder (BD) have an elevated risk of dementia, but the underlying etiology is unclear. Examining the genetic risk for Alzheimer's disease (AD) and dementia with Lewy bodies (DLB) is crucial to understanding this increased risk and uncovering underlying mechanisms, which are essential for improving patient outcomes.

**Method:**

We included 2,238 genotyped individuals with bipolar disorder (BD) from the Swedish Bipolar Collection (SWEBIC). Dementia diagnoses were identified using ICD‐10/9/8 codes from Swedish national patient register. *APOE* genotype was divided into *ε4* carriers and non‐carriers. Non‐APOE polygenic score (PGS) for AD (Bellenguez, 2022) and DLB (Chia, 2021) was calculated using PRS‐CS. In BD patients, Cox regression analyses examined PGS and *APOE ε4* carriership in relation to dementia risk, adjusted for age, sex, and ancestry (*N* dementia=76, *N* controls=2162).

**Result:**

A total of 76 participants developed dementia (mean age of 71.5 years, range: 53–84 years). Among individuals with bipolar disorder, *APOE ε4* carriership (HR: 1.88, 95% CI: 1.19–2.98, *p* = 0.007) and AD‐PGS (HR: 1.34, 95% CI: 1.06–1.68, *p* = 0.01) were associated with an increased risk of dementia. However, DLB‐PGS was not associated with risk of dementia (HR: 1.17, 95% CI: 0.91‐1.47, *p* = 0.2).

**Conclusion:**

The findings indicate that genetic risk for AD contributes to dementia development in individuals with bipolar disorder. These results provide insights into the etiology of dementia in bipolar disorder, highlighting the genetic interplay between the two conditions and informing strategies for risk stratification and targeted prevention.